# The cutoff point of clinical chronic obstructive pulmonary disease questionnaire for more symptomatic patients

**DOI:** 10.1186/s12890-018-0601-0

**Published:** 2018-02-27

**Authors:** Yong Suk Jo, Sangshin Park, Deog Kyeom Kim, Chul-Gyu Yoo, Chang-Hoon Lee

**Affiliations:** 1 Division of Pulmonary and Critical Care Medicine, Department of Internal Medicine, Seoul National University Hospital, 101 Daehak-Ro Jongno-Gu, Seoul, 03080 Republic of Korea; 2 Center for International Health Research, Rhode Island Hospital, The Warren Alpert Medical School of Brown University, Providence, RI 02903 USA; 3 Department of Pediatrics, The Warren Alpert Medical School of Brown University, Providence, RI 02903 USA; 4 Division of Pulmonary and Critical Care Medicine, Department of Internal Medicine, Seoul Metropolitan Government-Seoul National University Boramae Medical Center, Seoul, Republic of Korea

**Keywords:** Chronic Obstructive Pulmonary Disease, Correlation study, Health related quality of life, Clinical Questionnaires

## Abstract

**Background:**

An adequate threshold for the Clinical Chronic Obstructive Pulmonary Disease (COPD) Questionnaire (CCQ) defining more symptomatic COPD patients has not been determined. We aimed to determine the efficacy of the CCQ and the appropriate CCQ threshold for more symptomatic COPD patients.

**Methods:**

COPD patients aged > 40 years who smoked/had smoked ≥10 packs/year were prospectively enrolled over 1 year from three South Korean hospitals (*n* = 126). Correlations between the CCQ and St. George’s Respiratory Questionnaire (SGRQ), COPD Assessment Test (CAT), the modified Medical Round Council (mMRC) scale, lung function, and exercise capacity were evaluated. “More symptomatic patients” were those with an SGRQ score ≥ 25. Area under the receiver operating curve and classification and regression tree analyses were performed to determine the CCQ threshold equivalent to an SGRQ score ≥ 25.

**Results:**

The CCQ significantly correlated with the SGRQ, CAT, and mMRC scale (*r* = 0.76, 0.69, and 0.53, respectively). A CCQ cutoff of 1.4 predicted an SGRQ score of 25 better than others. A CCQ score of 1.4 was a significant determinant of an SGRQ score ≥ 25 even after adjusting for potential confounders.

**Conclusions:**

The CCQ was correlated with other symptom indicators, lung function, and exercise capacity. A CCQ cutoff of 1.4 agreed better than CCQ cutoff of 1.0, suggested by guideline, and this cutoff value may identify more symptomatic COPD patients well.

**Trial registration:**

ClinicalTrials.gov Identifier: NCT02527486. Date of registration: December 19, 2014, retrospectively registered.

## Background

Chronic obstructive pulmonary disease (COPD), characterized by persistent and progressive airflow limitation, is one of the major causes of morbidity and mortality worldwide, and its prevalence is increasing. COPD was previously regarded as a disease mainly characterized by breathlessness, but recently it has been reported that COPD has multiple symptomatic effects on health-related quality of life (HRQOL) [1]. The severity of airflow limitation alone is not strongly correlated with HRQOL in COPD patients [2]; therefore, comprehensive methods to evaluate the COPD-specific health status are needed.

Since 2011, Global Initiative for Chronic Obstructive Lung Disease (GOLD) adopted St. George’s Respiratory Questionnaire (SGRQ) as a new assessment tool for COPD patients considering their current respiratory symptoms and future risk of exacerbation. Currently, GOLD emphasizes HRQOL more than dyspnea alone and divides patients into more symptomatic (SGRQ ≥25) and less symptomatic (SGRQ < 25) patients based on previous clinical trials of long-acting bronchodilators [3–5].

SGRQ is the most widely used and valid tool to evaluate the health status of COPD patients [6] and is composed of 50 items with 76 weighted responses. The SGRQ scoring method is a bit difficult considering the amount of items and weighted responses required, and many COPD patients may find it difficult to complete without any help [7]. Thus, the COPD Assessment Test (CAT) was developed to compensate for the complexity of SGRQ [8, 9], and many studies which have evaluated the distribution of patients of GOLD grading classification according to the tool used to assess symptoms, found a different proportion of patients in each category using CAT score ≥ 10. With this background, GOLD recommended that the equivalent point of CAT for SGRQ of 25 is 10 [8–12].

Clinical COPD Questionnaire (CCQ), which has a good correlation with SGRQ [13, 14], is also thought to be a good instrument for assessing health status in COPD patients and is simple to use [13]. However, although studies have reported that a CCQ score range of 1.0–1.5 is consistent with an SGRQ score of 25 [14], until now, a clear CCQ cutoff point that is equivalent to an SGRQ score of 25 has not been determined. Furthermore, to date, the CCQ has not been sufficiently validated for evaluating health status in COPD patients in Asia [15].

Therefore, the present study aimed (i) to assess whether the CCQ correlates well with other health status measures, lung function, and exercise capacity in Korean COPD patients, and (ii) to determine the CCQ cutoff point that corresponds well with the SGRQ cutoff point of 25, considered the standard representing more symptomatic patients.

## Methods

### Study design and participants

The Seoul National University Airway Registry is a prospective observational cohort study that enrolls patients with chronic airway disease, including COPD.(ClinicalTrials.gov Identifier: NCT02527486). Among the participants in the cohort, patients aged 40 years or more who were diagnosed as having COPD between April 2013 and December 2015 at Seoul National University Hospital, a tertiary care hospital, and between March 2014 and December 2015 at Boramae Medical center, a municipal hospital, and Seoul National University Bundang Hospital, were included in our study. A COPD case was defined as a current or former smoker (≥10 packs/year) with chronic respiratory symptoms who had a post-bronchodilator forced expiratory volume in 1 s (FEV_1_) to forced vital capacity ratio < 0.7. Participants who were not assessed either by SGRQ, the CAT, or the CCQ at the baseline visit were excluded. All patients signed the informed consent form to participate in this observational cohort. This study was approved by the Institutional Review Board (IRB) of Seoul National University Hospital (IRB no. 1602-122-743).

For COPD patients, a previous history of symptoms, exposure to smoking and biomass smoke, experiencing exacerbation, and comorbidities were evaluated at baseline. Pulmonary function tests including spirometry and lung volume measurements were performed at least annually. Spirometry was performed by standardized equipment, and lung volume was measured following the American Thoracic Society/European Respiratory Society guidelines [16, 17]. Spirometry was repeated at least three times to verify the reproducibility and validity, and assessments of the pulmonary function test results were performed using computer programs and reviewed by highly qualified physicians. The spirometric reference value was calculated by Morris’s predictive equation [18, 19].

Acute exacerbations were evaluated based on self-reported symptom aggravation requiring modification of current treatment. Severe exacerbation was defined as an acute exacerbation event requiring a visit to the emergency room or hospitalization, and moderate exacerbation was defined as an acute exacerbation requiring a visit to an outpatient clinic and treatment with short-term systemic corticosteroids or antibiotics. Our definition of exacerbation was mainly based on a modified respiratory questionnaire from the Epidemiology Standardization Project questionnaire [20].

### Clinical assessment instruments

The SGRQ total score ranges from 0 to 100 where 100 indicates the worst quality of life, and its minimum clinically important difference (MCID) value is 4 units [21]. The CAT total score ranges from 0 to 40, and 2.0 units is suggested as its MCID value [22]. The modified British Medical Research Council (mMRC) questionnaire total score ranges from 0 to 4 where 4 means most severe dyspnea, and an mMRC score ≥ 2 is used as a cutoff point for dividing patients with less dyspnea from those with more dyspnea [23]. The 6-min walk test was performed to measure exercise capacity and assessed according to the American Thoracic Society guidelines, and its MCID value is 30 m [24].

The CCQ consists of 10 questions distributed in three domains (symptom, functional state, and mental state) assessed by a seven-point scale from 0 to 6, which indicate the best (asymptomatic and no limitation) and the worst conditions (extremely symptomatic and limited), respectively [13]. The CCQ total score is calculated by summing the scores of the 10 questions and dividing it by the number of items [25]. The Korean version of the CCQ was used in the present study [26].

### Statistical analysis

Descriptive data are expressed as mean ± standard deviation or number (%) unless otherwise specified. To evaluate the correlation between questionnaires (CCQ vs. SGRQ, CCQ vs. CAT, and CCQ vs. mMRC) and between CCQ and lung function indexes (FEV_1_% and 6-min walk distance), we used Pearson correlation analyses. The degree of agreement between two indexes is expressed as the correlation coefficient, r, where a positive value means positive correlation and the higher the value, the stronger the correlation.

We used several CCQ cutoff scores based on an SGRQ score of 25 and a CAT score of 10, to indicate more symptomatic patients according to guidelines. We also applied a CCQ score that is equivalent to an SGRQ score of 20, which is better than suggesting an SGRQ score of 25 for a CAT score of 10 [27]. To determine which CCQ cutoff score was the most equivalent to an SGRQ score of 25, an area under the receiver operating curve (AUROC) analysis was performed. A classification and regression tree (CART) analysis, a type of decision tree methodology for the identification of mutually exclusive subgroups of at-risk persons who share common characteristics related to particular health-related behavior, was also performed. Then, the Cohen’s kappa coefficient was calculated to compare the degree of agreement between the CCQ cutoff points and an SGRQ score of 25.

All analyses were two-sided and performed at a significance level of 0.05 unless otherwise noted. A *P*-value <.05 was considered statistically significant. All analyses were carried out using STATA version 13.1 (StataCorp, College Station, Texas, USA).

## Results

### Patient characteristics

**Fig. 1:**
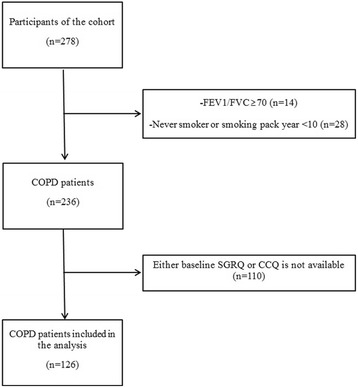
Patient disposition. Abbreviations: CCQ, Clinical COPD Questionnaire; COPD, chronic obstructive pulmonary disease; FEV_1_, forced expiratory volume in 1 s; FVC, forced vital capacity; SGRQ, St. George’s Respiratory Disease Questionnaire

Among 278 patients enrolled in the prospective cohort, we excluded 42 patients based on their spirometry and smoking pack/year and 110 patients whose health status indexes were not recorded (Fig. 1). Consequently, 126 patients aged 40 years or older who answered the questionnaires and agreed to regular follow-up at an outpatient clinic were finally enrolled.

**Fig. 2:**
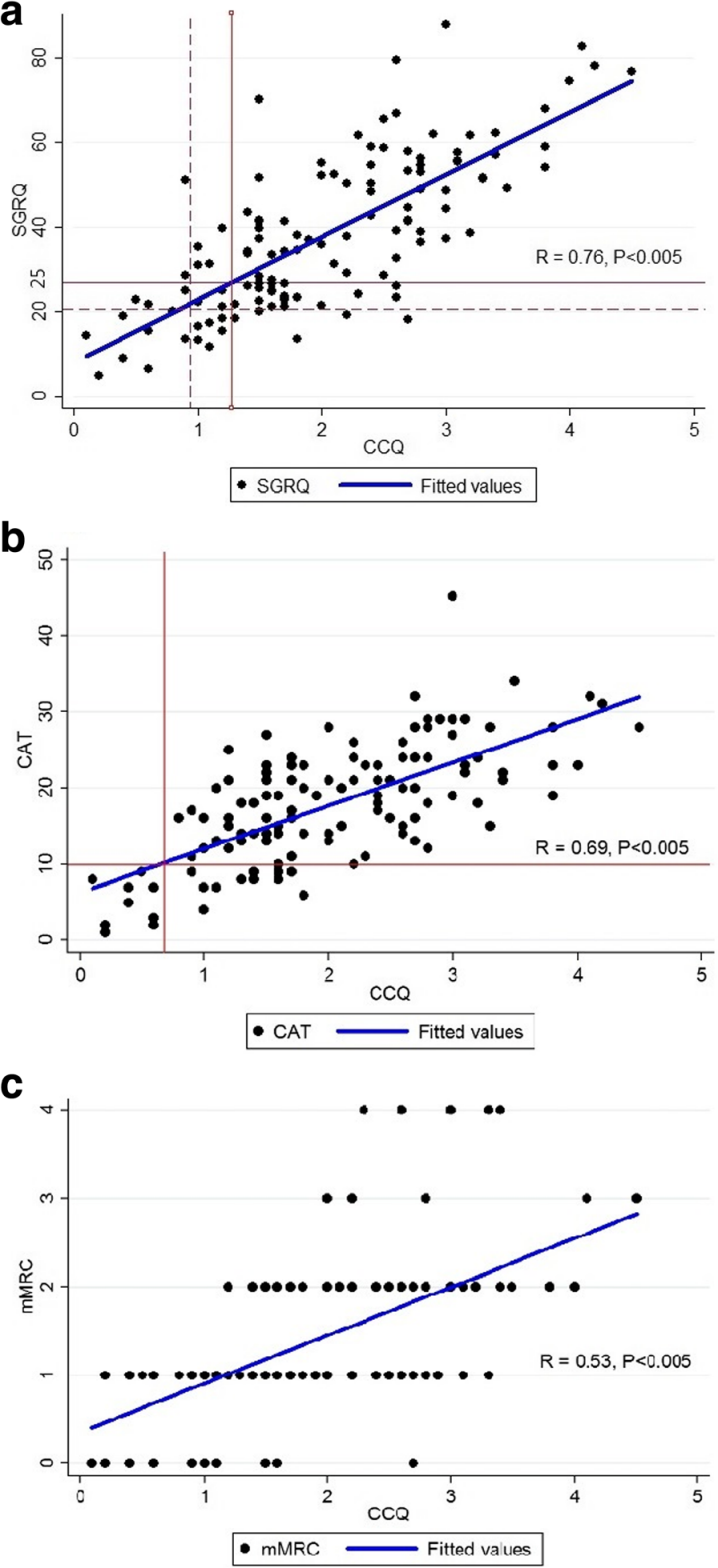
Correlation between the CCQ and the (**a**) SGRQ, (**b**) CAT, and (**c**) mMRC. **a**, **b** CCQ value for SGRQ score of 25, 20 and for CAT score of 10 determined by an area under the receiver operating curve (AUROC) analysis

The mean SGRQ, CAT, and CCQ scores were 38.04 ± 18.18, 17.76 ± 7.68, and 2.03 ± 0.94, respectively. Among 126 patients, 46 (36.51%) patients recalled that they experienced at least one acute exacerbation event 1 year before enrollment and 17 (13.49%) patients recalled more than two acute exacerbation events during the past year. The mean frequency of severe and moderate exacerbation was 1.4 ± 0.52 and 2.05 ± 2.01 events per year, respectively (Table 1). As can be seen in Figs. 2a and b, the CCQ values corresponding to the SGRQ scores of 25, 20, and a CAT score of 10 were 1.2, 1.0, and 0.7, respectively.

**Table 1:** Baseline characteristics of patients

Characteristics	*N* = 126
Age (years)	68.83 ± 7.23
Sex: Male	123 (97.62)
BMI (kg/m^2^)	22.97 ± 3.27
Smoking status	
Ex-smoker	88 (69.84)
Current smoker	38 (30.16)
Packs per/year	54.47 ± 64.46
History of exacerbations in the past year	
Unplanned visit to an outpatient clinic (no./year)	2.05 ± 2.01
Unplanned visit to an emergency department or hospital admission (no./year)	1.4 ± 0.52
≥ 2 events in the past year (n, %)	17 (13.49)
FEV1 (L)	1.65 ± 0.53
FEV1 (%)	65.35 ± 19.42
FVC (L)	3.59 ± 0.79
FVC (%)	97.15 ± 17.10
FEV1/FVC	45.95 ± 11.55
GOLD stage	
1 (FEV1 > 80% predicted)	23 (18.25)
2 (FEV1 50%–79% predicted)	76 (60.32)
3 (FEV1 30%–49% predicted)	23 (18.25)
4 (FEV1 < 30% predicted)	4 (3.17)
SGRQ score	38.04 ± 18.18
CAT score	17.76 ± 7.68
mMRC dyspnea score	1.44 ± 0.92
CCQ score	2.03 ± 0.94
6MWT (m)	444.39 ± 103.85

Data are presented as mean ± standard deviation or n (%)

*Abbreviations*: *BMI* body mass index, *CAT* Chronic Obstructive Pulmonary Disease Assessment Test, *CCQ* Clinical Chronic Obstructive Pulmonary Disease Questionnaire, *FEV1* forced expiratory volume in 1 s, *FVC* forced vital capacity, *GOLD* Global Initiative for Chronic Obstructive Lung Disease, *mMRC* modified Medical Research Council, *SGRQ* St. George’s Respiratory Disease Questionnaire, *6MWT* 6-min walk test

### Correlation between the CCQ and other health status questionnaires, lung function, and exercise capacity

**Fig. 3:**
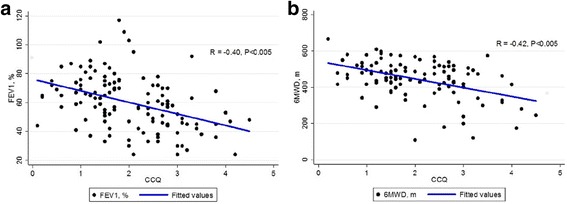
Correlation between the CCQ and the (**a**) FEV_1_% and (**b**) 6MWD. Abbreviations: CCQ, Clinical Chronic Obstructive Pulmonary Disease Questionnaire; FEV_1_, forced expiratory volume in 1 s; 6MWT, 6-min walk test

The CCQ significantly correlated well with SGRQ, the CAT, and the mMRC (r = 0.76, 0.69, and 0.53, respectively) (Fig 2). The CCQ showed a significant negative correlation with FEV_1_ and 6-min walk distance (r = − 0.40 and − 0.42, respectively) (Fig. 3).

### CCQ cutoff point to identify more symptomatic patients

**Fig. 4:**
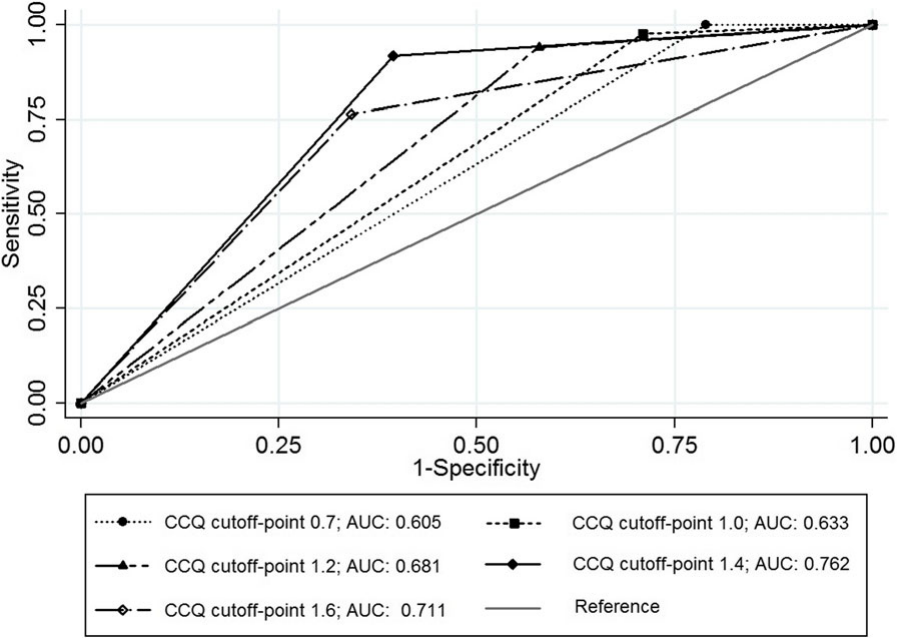
The area under the receiver operating curve to compare the concordance of SGRQ according to CCQ cut-off level. Abbreviations: AUC, area under curve; CCQ, Clinical Chronic Obstructive Pulmonary Disease Questionnaire; SGRQ, St. George’s Respiratory Disease Questionnaire

**Fig. 5:**
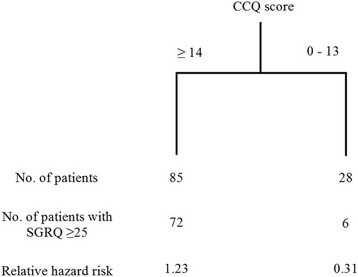
Classification and regression tree analysis of agreement with an SGRQ score ≥ 25 (adjusted by age, sex, body mass index, and FEV_1_). Abbreviations: FEV_1_, forced expiratory volume in 1 s; SGRQ, St. George’s Respiratory Disease Questionnaire

A cutoff point of 1.4 showed the highest AUROC for identifying more symptomatic patients with SGRQ ≥25 (AUROC = 0.605, 0.633, 0.681, 0.762, and 0.711 for the CCQ cutoff points of 0.7, 1.0, 1.2, 1.4, and 1.6, respectively) (Fig. 4). The categorization based on the CCQ cutoff point of 1.4 showed the highest agreement with the SGRQ 25-based categorization (CCQ cutoff point = 0.7: agreement rate = 75.61% and κ value = 0.27; CCQ cutoff point = 1: agreement rate = 76.42% and κ value = 0.33; CCQ cutoff point = 1.4: agreement rate = 82.11% and κ value = 0.56) (Table 2). Even when adjusting for age, sex, body mass index, and FEV_1_, a CCQ score ≥ 1.4 was the good determinant of an SGRQ score ≥ 25 in the CART analysis (relative hazard risk, 1.23) (Fig. 5).

**Table 2:** Agreement between SGRQ and the CCQ based on the new GOLD classification of the CCQ cutoff value

	CCQ < 1	CCQ ≥1
SGRQ < 25	11	27
SGRQ ≥25	2	83
Agreement: 76.42%; kappa value, 0.33		
	CCQ < 1.4	CCQ ≥1.4
SGRQ < 25	23	15
SGRQ ≥25	7	78
Agreement: 82.11%; kappa value, 0.56		
	CCQ < 0.7	CCQ ≥0.7
SGRQ < 25	8	30
SGRQ ≥25	0	85
Agreement: 75.61%; kappa value, 0.27		

*Abbreviations*: *CCQ* Clinical Chronic Obstructive Pulmonary Disease Questionnaire, *GOLD* Global Initiative for Chronic Obstructive Lung Disease, *SGRQ* St. George’s Respiratory Disease Questionnaire

## Discussion

This study demonstrated that the CCQ is well correlated with other health status measurements including SGRQ, pulmonary function, and exercise capacity in Korean COPD patients. In our study, based on AUROC and CART analyses, the CCQ cutoff point that best corresponds to an SGRQ score of 25 was 1.4, which showed a better agreement rate and a higher classification power compared with other cutoff points.

GOLD recommends a comprehensive approach to assess and adequately manage symptoms in COPD patients and proposes the mMRC, CAT, and CCQ as interchangeable instruments for evaluation of health status. However, despite the potential clinical importance, the CCQ has not been widely used in clinical practice because there is no known precise CCQ cutoff point that correlates with an SGRQ cutoff point of 25 (considered the standard marker of more symptomatic patients) and also CAT has been preferred than CCQ because CAT is more widely implemented and recommended by GOLD. GOLD guidelines recently set a CCQ score range of 1.0–1.5 as being equivalent to an SGRQ score of 25 [23], and recent studies suggest that the MCID value for the CCQ is approximately 0.4 [28, 29]. However, this wide range seems to be impractical for use in the clinical setting. In addition, GOLD tends to focus more on the CAT rather than the CCQ.

Based on the findings of the present study, we suggest that a CCQ cutoff point of 1.4 is appropriate for dividing COPD patients into more symptomatic and less symptomatic patients. This was based on two analyses. First, the AUROC value for a CCQ cutoff point of 1.4 was the highest for agreement with an SGRQ score of 25. Second, a CCQ cutoff point of 1.4 was the best value to distinguish between an SGRQ score ≥ 25 and < 25 in the CART analysis. Consequently, a CCQ cutoff point of 1.4 was more concordant with an SGRQ cutoff point of 25, when compared to a CCQ cutoff point of 1.0. A low agreement was found with the SGRQ score of 25, when analysis was performed based on the CCQ cutoff point of 0.7, which corresponds to the CAT score of 10, based on CAT instead of SGRQ. This is in line with our previous report, which states that scores higher than a CAT score of 10, say 15, are more useful for assessment of the severity of symptoms [30].

SGRQ is the most comprehensive disease-specific health status measure for COPD patients. However, because it is composed of 50 items, it is complicated for older COPD patients to answer without any help, and it takes a relatively long time to complete [7]. The CCQ is thought to be an alternative to SGRQ and consists of only 10 questions assessed by a seven-point scale from 0 to 6. Because the CCQ is a simple tool, it takes about 2 min to complete, and subjects require no specific help to complete it [13]. Along with its simplicity, the CCQ is a comprehensive tool to evaluate the health status in COPD patients because it has three domains (symptoms, functional state, and mental state), which means that the CCQ has psychometric properties that make it more useful [13]. CAT, one of the symptom and HRQOL measurement proposed by GOLD, is one-dimensional tool, while CCQ is similar to SGRQ, which is the most comprehensive tool. Further, CCQ is more simple and easy to use than SGRQ. Since it contains the description for each score, patients can sassily respond to the questions by themselves. In this aspect, the present study is focused on CCQ.

This study confirmed the clinical efficacy of the CCQ for evaluating the health status of COPD patients. The CCQ correlates well with other health status measures, lung function, and exercise capacity. In addition, recent studies have suggested that an upward revised CAT as a symptom threshold could reflect health status more accurately than the existing CAT cutoff point (≥10), which was previously regarded as a surrogate of the SGRQ [30–32]. Furthermore, our study determined a new and clear CCQ threshold for categorizing COPD patients as more or less symptomatic.

The present study has several limitations to consider. First, we conducted a cross-sectional analysis and did not investigate the predictive value of CCQ for lung function decline or future exacerbation risk. Second, although the CCQ consists of three domains (symptoms, functional state, and mental state), we did not record the three subcategories separately, but rather evaluated the CCQ as a whole. Third, only small number of women were included because of the low prevalence of female smokers, therefore there may be a limitations in external validity. Fourth, the gold standard of cutoff points to measure symptoms in COPD remained open for discussion. Thus, we also showed results of applying the CAT score of 10 as a standard for the symptom criteria. Lastly, the suggested CCQ cutoff point of 1.4 needs to be externally validated.

## Conclusions

The CCQ showed significant clinical value for assessing the health status of patients with COPD and showed good correlation with other questionnaires including SGRQ and the CAT as well as lung function and exercise capacity. Although the gold standard tool for assessing the severity of symptom in COPD patients may still be debated, CCQ threshold 1.4 showed a better agreement with SGRQ threshold of 25, suggested by GOLD.

## Abbreviations

6MWT: 6-min walk test
BMI: Body mass index;
CAT: Chronic Obstructive Pulmonary Disease Assessment Test
CCQ: Clinical Chronic Obstructive Pulmonary Disease Questionnaire
FEV_1_: Forced expiratory volume in 1 s
FVC: Forced vital capacity
GOLD: Global Initiative for Chronic Obstructive Lung Disease
mMRC: Modified Medical Research Council
SGRQ: St. George’s Respiratory Disease Questionnaire
